# Determination of Finger Optical Properties Using an Integrating Sphere

**DOI:** 10.3390/s26072173

**Published:** 2026-03-31

**Authors:** Markus Wagner, Benedikt Beutel, Peter Naglic, Oliver Fugger, Florian Foschum, Alwin Kienle

**Affiliations:** 1 Faculty of Natural Sciences, Ulm University, D-89081 Ulm, Germany; 2 Institute for Laser Technologies in Medicine and Metrology at the University of Ulm, Helmholtzstr. 12, D-89081 Ulm, Germany

**Keywords:** integrating sphere, finger phantom, cylinder, optical properties, tissue components, silicone, oxygenation

## Abstract

Integrating sphere measurements are a well-established method for determining the optical properties of planar samples. In this study, the approach was expanded from slab geometry to cylindrical geometry illuminating the cylinder barrel, thereby demonstrating its applicability for determining the optical properties of human fingers. By adapting existing integrating sphere theory to cylindrical samples, the method was systematically validated using phantoms and subsequently applied to human fingers. It has been demonstrated that the absorption coefficient $\mu_{a}$ and the reduced scattering coefficient $\mu_{s}^{\prime }$ of cylindrical and 3D finger phantoms can be determined with a high degree of agreement to those of slab phantoms. Moreover, this approach facilitates the quantification of tissue components in human fingers, including fat, water and collagen content, total haemoglobin concentration and tissue oxygenation.

## 1. Introduction

The knowledge of the optical properties of tissue is necessary to develop optical medical diagnostic and therapeutic techniques. A variety of measurement techniques are employed to ascertain the optical properties of tissue and thereby quantify its components. Such techniques include systems in the time domain, spatial frequency domain, spatial domain and integrating sphere systems [1]. The integrating sphere is typically used to measure the optical properties of different ex vivo biological samples, namely their absorption coefficient $\mu_{a}$ and the reduced scattering coefficient $\mu_{s}^{\prime }$ (also named as effective scattering coefficient) [2,3,4,5]. These data are widely used as reference data. Integrating sphere systems are an established method for determining the optical properties of various materials based on the measured, angle-independent reflectance *R* and transmittance *T*. To date, these measurements and the associated analytical and numerical models have been based predominantly on planar samples (slabs) [6,7,8,9,10,11,12]. To determine the optical properties $\mu_{a}$ and $\mu_{s}^{\prime }$ with an integrating sphere, it is sufficient to measure *R* and *T* from a slab. In vivo optical properties have not yet been determined with the integrating sphere mainly due to the involved more complicated geometry. The finger, e.g., involved in PPG (photoplethysmography) measurements, represents an important measurement position, but does not correspond to the slab geometry used for integrating sphere measurements. Alternative geometries must therefore be developed and evaluated. In this study, alternative geometries are performed using optical phantoms in slab, cylinder and finger shapes to demonstrate their applicability for in vivo measurements.

Optical phantoms are materials used in biomedical optics to replicate the optical properties of biological tissue. These phantoms can be fabricated from various base materials, including epoxy resin and silicone. The optical properties of these materials, specifically the absorption coefficient and reduced scattering coefficient, can be precisely tuned by incorporating scattering particles or absorbing media. Various fabrication approaches for optical phantoms have been documented in the literature [13,14,15,16]. Comprehensive reviews of phantom types in biomedical imaging and their fabrication criteria are provided by Pogue et al. [17] and Hacker et al. [18].

In [16], our group demonstrated that the cylindrical model is applicable for fingers by using a phantom that mimicked the reflectance and transmittance properties of a human finger in the visible range. However, this previous work lacked a systematic investigation of the accuracy, utilised only the visible wavelength range and did not determine tissue components of different fingers. These limitations are addressed in the present work.

The objective of this work was to systematically investigate the applicability of integrating sphere measurements for cylindrical samples and human fingers to determine the absorption coefficient $\mu_{a}$ and reduced scattering coefficient $\mu_{s}^{\prime }$. To achieve this, an evaluation model based on cylindrical geometry was developed and integrated into the measurement framework. The proposed model was validated through systematic comparisons of optical properties measured across a broad wavelength range using a comprehensive set of tissue-mimicking phantoms, including both slab and cylindrical geometries of varying thicknesses, followed by evaluation with finger phantoms. Subsequently, measurements on human fingers were conducted to determine their optical properties $\mu_{a}$ and $\mu_{s}^{\prime }$ assuming a homogeneous medium. The derived absorption coefficient was further used to quantify tissue components, such as fat, water, collagen, oxygenated and deoxygenated haemoglobin.

## 2. Materials and Methods

### 2.1. Phantom Material and Manufacturing Process

**Figure A1:**
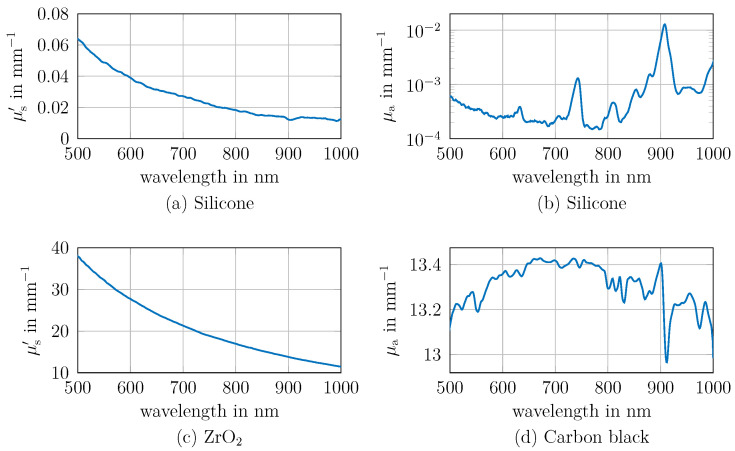
Optical properties $\mu_{s}^{\prime }$ and $\mu_{a}$ of the used materials. (**a**) shows $\mu_{s}^{\prime }$ of the used silicone according to [16]. The absorption of the silicone (**b**) was determined newly compared to [16]. (**c**) shows $\mu_{s}^{\prime }$ of the used zirconium oxide preconcentration, $\mu_{a}$ is neglected. (**d**) depicts the absorption of the carbon black in the prediluted concentration, $\mu_{s}^{\prime }$ is neglected due to low concentrations used.

The silicone base material was Elastosil M 4641 A/B (Wacker Chemie AG, München, Germany). Zirconium oxide (ZrO_2_) nanoparticles 200 nm (US Research Nanomaterials Inc., Houston, TX, USA) were employed to adjust the scattering properties of the phantom. As an absorber, carbon black (Lampenruß, Guardi Feinste Künstlerpigmente, Wien, Austria) was used. The manufacturing process used to produce the phantoms is more precisely described in [16]. Shortly, the whole mixing process is based on a multi-step mixing procedure combined with a double asymmetric vacuum centrifuge (SpeedMixer DAC 800, Hauschild Engineering, Hamm, Germany). To create the phantoms, the pigments (carbon black (CB) and ZrO_2_ particles) were first preconcentrated in component A of the silicone to ensure reproducible $\mu_{a}$ and $\mu_{s}^{\prime }$. The wavelength-dependent absorption coefficient $\mu_{a}$ and the reduced scattering coefficient $\mu_{s}^{\prime }$ of the preconcentrations, shown in Figure A1, were determined by preparing test slabs with different pigment concentrations. $\mu_{a}$ and $\mu_{s}^{\prime }$ of the test slabs were determined with an integrating sphere setup. The resulting $\mu_{a}$ and $\mu_{s}^{\prime }$ of the preconcentration were calculated by subtracting $\mu_{a}$ and $\mu_{s}^{\prime }$ of the control sample from pigmented samples and dividing by the pigment concentration [16]. The intrinsic pigment scattering of carbon black was neglected due to the low concentrations used. Additionally, no measurable absorption of the ZrO_2_ particles was detected and was therefore neglected. The $\mu_{s}^{\prime }$ values of the silicone were taken from [16], while the $\mu_{a}$ of the silicone was newly determined for this study.

With the knowledge of $\mu_{a}$ and $\mu_{s}^{\prime }$ of the preconcentrations and silicone, the needed concentrations were calculated to achieve the aimed $\mu_{a}$ and $\mu_{s}^{\prime }$ of the phantoms at one wavelength. After weighting, the components are mixed and poured into moulds according to [16].

### 2.2. Moulding

**Figure 1:**
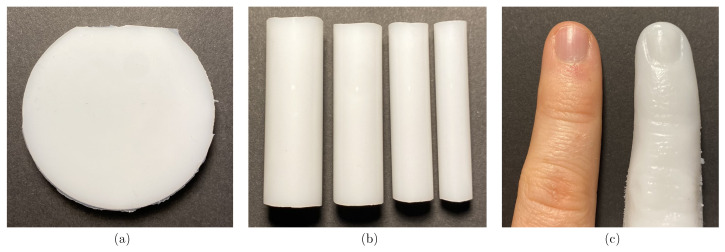
Three different phantom geometries were made from the same pigmented silicone material: (**a**) A slab phantom with a diameter of 50 mm and a thickness of 6 mm and 8 mm. (**b**) Four cylindrical phantoms with diameters of 14 mm, 12 mm, 10 mm and 8 mm (**left** to **right**). (**c**) A finger phantom (**right**) compared to the real finger (**left**), which was used as a model. The phantoms presented here are referred to as Phantom P1 later in the paper.

To evaluate the usability of our integrating sphere measurement system for a different geometry than a slab geometry, the same pigmented silicone was poured into three different phantom geometries: slabs, cylinders and a realistic finger shape; see Figure 1.

The slabs had different thicknesses (6 and 8 mm) and a diameter of 50 mm. The slabs were made by pouring the pigmented silicone between two acrylic glass plates in a 3D-printed mould (Pro3, Raise3D Technologies, Irvine, CA, USA), following the method previously reported by Wagner et al. [16]. These slabs were subsequently used to determine the optical properties of the phantom material. The cylinder phantoms with a diameter of 8, 10, 12 and 14 mm and a height of 40 mm were fabricated by pouring the pigmented silicone into acrylic glass pipes with the corresponding internal diameter (diconfa GmbH, Pattensen, Germany).

To ensure production of reproducible finger phantoms, the pigmented silicone was poured into a custom 3D-printed finger mould with anatomically realistic geometry. The 3D-printed finger mould of an existing human finger was made in a three-step process. Firstly, a silicone phantom of a real finger was made by curing silicone in an alginate finger mould (TFC Alginat PREMIUM, Trollfactory, Riede, Germany) in order to minimise movement artefacts when determining the 3D shape using a 3D scanner. A real finger served as the template for this process. Secondly, a 3D model of the finger phantom was made using a 3D scanning sensor (COMET®, ZEISS, Oberkochen, Germany). Thirdly, a two-piece mould was created using this 3D model and printed using a stereolithography (SLA) printer (Sonic Mighty Revo 14K, Phrozen, Hsinchu, Taiwan).

### 2.3. Integrating Sphere Measuring Setup and Inverse Model

The reflectance, transmittance and optical properties $\mu_{a}$ and $\mu_{s}^{\prime }$ of the samples were characterised using an integrating sphere-based measurement system developed by Foschum et al. and Bergmann et al., a laboratory version of the SphereSpectro 150H (Gigahertz Optik GmbH, Munich, Germany) [6,7]. The integrating sphere setup consists of a 3D-printed integrating sphere with a barium sulfate-coated interior and an inner diameter of 150 mm. For this study, the wavelength range from 400 nm to 1000 nm was used. The evaluation process determines the absorption coefficient $\mu_{a}$ and the reduced scattering coefficient $\mu_{s}^{\prime }$ from slab samples and is based on Monte Carlo simulations, a numerical solutions of the radiative transport equation, for volume scattering. The Monte Carlo results are combined with an analytical model used for the light propagation in the integrating sphere [6]. For all measurements, the asymmetry factor *g* was set to 0.75 for all wavelengths, assuming a Henyey–Greenstein scattering phase function since only optically thick samples were measured, for which the exact phase function has a minor impact. The wavelength-dependent refractive index of the silicone material, ranging from 1.43 to 1.40 across the visible spectrum, was determined via ellipsometry (SENresearch 4.0, SENTECH Instruments GmbH, Berlin, Germany) as reported by [16]. All measurements were performed in triplicate to ensure reproducibility. Sample thickness at the measurement location was precisely determined using a micrometer screw serving as the cylinder diameter in the model for cylinders and fingers.

**Figure A2:**
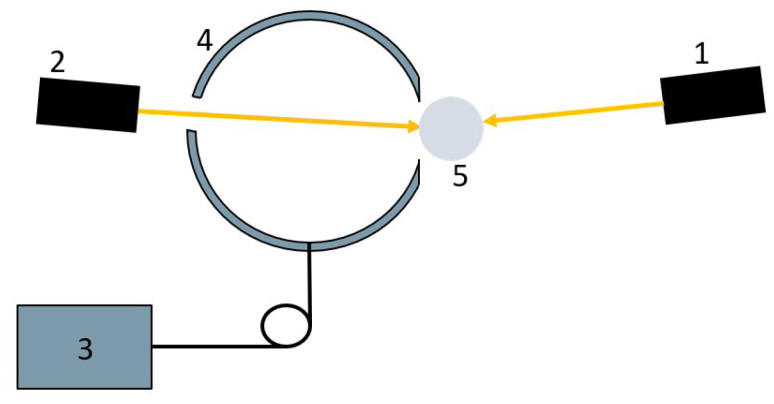
Schematic top view of the integrating sphere setup for measurements on cylindrical samples. (1) Light source for transmittance, (2) light source for reflectance, (3) spectrometer, (4) integrating sphere and (5) cylindrical sample or finger.

To determine $\mu_{a}$ and $\mu_{s}^{\prime }$ of cylindrical samples including human fingers, the geometry in the Monte Carlo simulation was adapted and a customised look-up table (LUT) was generated. The cylinder and finger were modelled as a homogeneous cylinder with perpendicular illumination onto the cylinder barrel in the Monte Carlo simulation. Figure A2 illustrates the schematic setup for the integrating sphere measurements on both the cylindrical geometries and fingers. The light source in the Monte Carlo simulation was modified to accurately replicate the optical imaging characteristics of the actual light sources used concerning its direction, diameter and numerical aperture. The photons exiting the cylinder were tracked in the simulation to determine whether they entered the integrating sphere through its circular port. Based on these simulations and the measured *R* and *T* of a cylinder, $\mu_{a}$ and $\mu_{s}^{\prime }$ were determined.

At wavelengths where transmittance values become too small, reflectance measurements alone create ambiguity in determining $\mu_{a}$ and $\mu_{s}^{\prime }$. To address this limitation, a modified evaluation approach was implemented that calculates $\mu_{a}$ while utilising an extrapolated $\mu_{s}^{\prime }$ and a measured *R*. The reduced scattering coefficient $\mu_{s}^{\prime }$ was determined from 400 nm to 1000 nm using the power law equation, given by Equation (2), with the corresponding coefficients. Based on these $\mu_{s}^{\prime }$ and the measured reflectance, the absorption coefficient was subsequently computed employing the LUT.

### 2.4. Main Tissue Components and Analysis

**Figure 2:**
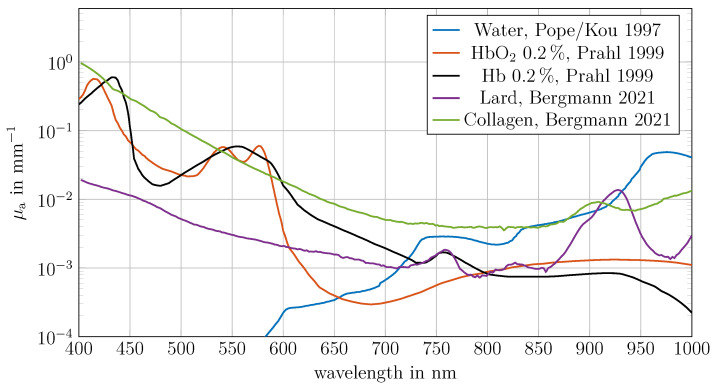
Absorption coefficients of the tissue components used for human tissue were obtained from the following sources: blood data from Prahl [19], collagen and fat data from Bergmann et al. [2], and water data from Pope and Kou [20,21].

The obtained spectral absorption coefficient of the human fingers were compared to the spectral absorption coefficients of tissue components. The main contributors of absorption in human tissue between 650 nm and 1000 nm are deoxygenated haemoglobin (Hb), oxygenated haemoglobin (HbO_2_), water, lipids, melanin, collagen and elastin [2]. For blood, data from Prahl [19], for collagen and fat, data from Bergmann et al. [2] and for water, data from Pope and Kou [20,21] were used. Elastin was neglected due to its high spectral similarity to collagen between 650 nm and 1000 nm. No differentiation was made between the spectra of haemoglobin and myoglobin. All test subjects were classified as Fitzpatrick type 1. Melanin was omitted from the analysis because the fitting was conducted in the 650 nm to 1000 nm range, where its contribution is minimal, particularly for low Fitzpatrick types. The absorption data used are shown in Figure 2.

The total absorption of the considered tissue is composed of the tissue chromophores’ absorption spectra times their volume fractions. If the absorption spectra of all components are known, the absorption coefficient can be described as $\mu_{a}\left(\lambda \right)=\sum c_{v,\mathrm{comp},n}\cdot \mu_{a,\mathrm{comp},n}\left(\lambda \right)$, where $c_{V,\mathrm{comp}}$ is the volume concentration of the tissue component *n* and $\mu_{a,\mathrm{comp}}$ is the wavelength-dependent absorption coefficient of the tissue component *n*. To determine the volume concentrations, a nonlinear regression (fmincon, MATLAB R2023a, MathWorks, Natick, MA, USA) was performed by minimising the weighted least squares of the measured absorption spectra for each finger, considering wavelengths from 650 nm to 1000 nm. The limits and initial parameters of the fitting algorithm have been constrained to the range between 0 and 1. Additionally, the fitting process was repeated 30 times with random initial parameters and the results with the smallest $\chi^{2}$ was chosen. The deviations between the fitted spectrum $\mu_{a,\mathrm{fit},i}$ and the measured values $\mu_{a,\mathrm{Meas},i}$ were quantified with the error metric *E*, the following equation was used:

(1) $$E=\frac{1}{N-P}\sum_{i=1}^{N}{\left(\frac{\mu_{a,fit,i}-\mu_{a,Meas,i}}{\sigma_{\mu_{a,Meas,i}}}\right)}^{2},$$

where N=269 is the total number of spectral values, P=5 is the number of parameters of the fit model and $\sigma_{\mu_{a,Meas,i}}$ is the standard deviation of three measurements of the measured absorption coefficient [22]. Furthermore, the goodness of fit was evaluated by calculating the coefficient of determination $R^{2}$.

In addition to the absorption coefficient, tissue can also be characterised by the reduced scattering coefficient. To model this, the reduced scattering coefficient was fitted to a power law with the coefficients *a* and *b*:

(2) $$\mu_{s}^{\prime }=a\cdot {\left(\frac{\lambda }{\lambda_{0}}\right)}^{-b},$$

where $\lambda_{0}=600\;\mathrm{nm}$. As described in [23], tissue scattering cannot be fully represented by a simple power law (*a* and *b*) alone but also includes a $c\cdot \lambda^{-3}$ term, which accounts for scattering caused by long cylindrical scatterers, such as collagen fibres. Thus, this scattering contribution increases with decreasing wavelength. However, since $\mu_{s}^{\prime }$ was only determined down to 650 nm in this study, the $\lambda^{-3}$ term was neglected for the shown results. Rayleigh scattering was also excluded due to the considered wavelength range. The parameters *a* and *b* of this model are directly related to the density, size and shape of the scatterers within the medium [24].

## 3. Results and Discussion

### 3.1. Optical Properties of Slab Phantoms

Three sets of optical phantoms were fabricated incorporating different concentrations of carbon black and zirconium oxide. The phantoms were designed with reduced scattering coefficients of approximately 1, 1.5 and 2 mm^−1^ at 700 nm, while maintaining an absorption coefficient of approximately 0.02 mm^−1^ at 700 nm, consistent with expected values for biological tissue. In the following, the phantoms were named P1 ($\mu_{s}^{\prime }$ = 1 mm^−1^, $\mu_{a}$ = 0.02 mm^−1^), P2 ($\mu_{s}^{\prime }$ = 1.5 mm^−1^, $\mu_{a}$ = 0.02 mm^−1^) and P3 ($\mu_{s}^{\prime }$ = 2 mm^−1^, $\mu_{a}$ = 0.02 mm^−1^).

The phantoms were prepared according to the methodology described in Section 2.1. First, a preconcentration of both the carbon black ($\mu_{a}$(700 nm) = 13.4 mm^−1^) and the zirconium oxide ($\mu_{s}^{\prime }$(700 nm) = 21 mm^−1^) was made. The resulting $\mu_{a}$ and $\mu_{s}^{\prime }$ of the preconcentration are shown in Figure A1. The specific concentrations of the preconcentrations used in the phantoms were as follows: P1 c_m, CB_ = 0.001654 g/g, c_ZrO_2__ = 0.047607 g/g; P2 c_m, CB_ = 0.001611 g/g, c_ZrO_2__ = 0.071418 g/g; P3: c_m, CB_ = 0.001671, c_ZrO_2__ = 0.095979.

**Figure 3:**
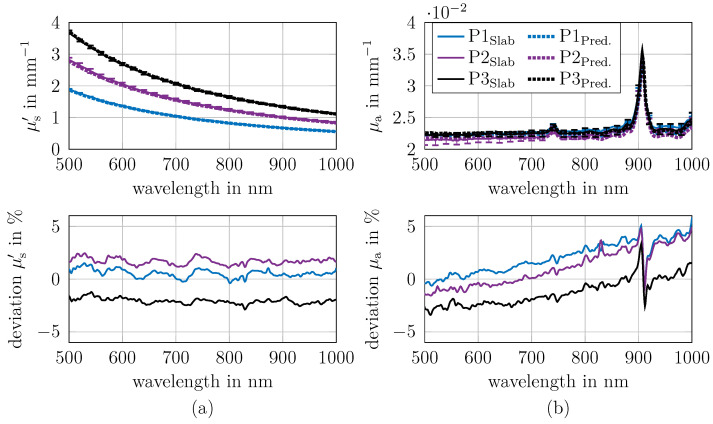
Optical properties $\mu_{a}$ and $\mu_{s}^{\prime }$ of phantoms P1, P2 and P3 determined from slabs using an integrating sphere. For comparison, predicted values are shown as dashed lines and are calculated from the mass concentrations of the used materials (raw spectra are shown in Figure A1). The (**top row**) shows the measured and the predicted $\mu_{s}^{\prime }$ (panel (**a**)) and $\mu_{a}$ (panel (**b**)). The (**bottom row**) shows the corresponding deviation between prediction and measurement. The maximum mean error between prediction and measurement across all wavelengths is 2% for both $\mu_{a}$ and $\mu_{s}^{\prime }$.

The optical properties $\mu_{a}$ and $\mu_{s}^{\prime }$ of the fabricated phantoms were determined with the integrating sphere setup and are presented in Figure 3 (top row, solid line). Therefore the slab phantoms (example photo; see Figure 1a) are used with the thickness 8 mm for P1 and 6 mm for P2 and P3. The slabs thickness were adjusted to get the optimal *R* and *T* values.

To verify the plausibility of the determined $\mu_{a}$ and $\mu_{s}^{\prime }$, the determined values were compared with theoretical predictions derived from the known concentrations of the materials used. The theoretical prediction is based on the intrinsic values of the $\mu_{a}$ and $\mu_{s}^{\prime }$ of the phantom components. The intrinsic $\mu_{a}$ and $\mu_{s}^{\prime }$ of the silicone were also considered, as shown in Figure A1. The deviations between prediction and measurement is shown in the bottom row of Figure 3, and in the top row, the predicted values are depicted (dashed line).

The mean deviation between prediction and measurement is below 2% for $\mu_{a}$ and $\mu_{s}^{\prime }$. The maximum deviation in $\mu_{a}$ is around 5%, and around 2% in $\mu_{s}^{\prime }$. These findings confirm both the accuracy of the phantom preparation protocol and the reproducibility of the measurement methodology. The oscillating errors in $\mu_{s}^{\prime }$ are due to the finite size of the used look-up table and the linear interpolation.

In future, it is proposed that the reduction in the oscillating errors could be addressed by utilising a neural network as an alternative to a look-up table. In $\mu_{a}$, the deviations varies with wavelength, primarily due to the absorption of silicone, which is challenging to determine accurately because of its low values. In general, the absolute deviations in $\mu_{a}$ between predictions and measurements is approximately 0.001 mm^−1^, which is sufficiently small. The examined wavelength range is limited to the spectrum between 500 nm and 1000 nm due to the decreased signal noise ratio of the transmittance for cylinders of 12 mm and 14 mm outside this wavelength range.

### 3.2. Comparison of the Optical Properties Between the Cylinder and Slab Phantoms

**Figure 4:**
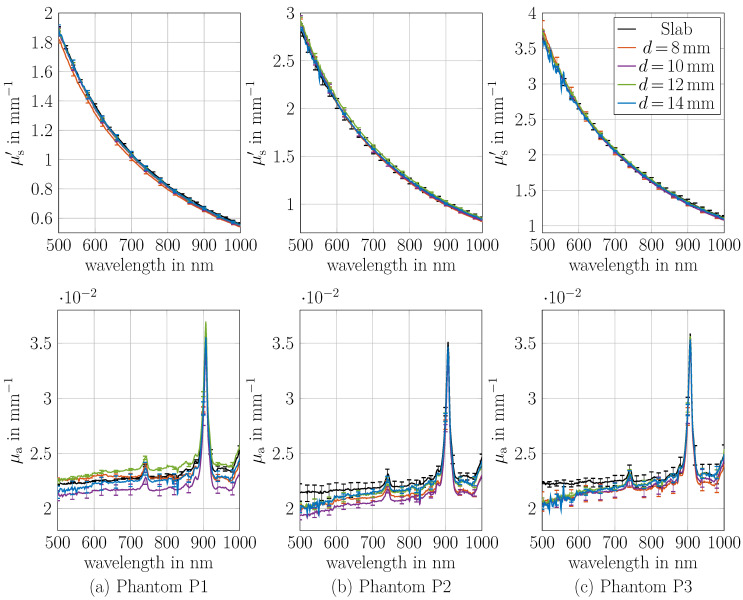
Optical properties $\mu_{a}$ and $\mu_{s}^{\prime }$ of cylinders with diameters of 8, 10, 12 and 14 mm compared to those of a slab made from the same material. Phantoms P1, P2 and P3 are shown in Panel (**a**–**c**), respectively. The maximum deviations compared to the measurement on the slab geometry over all wavelengths is 6.5% in $\mu_{a}$ and 3.5% in $\mu_{s}^{\prime }$. A picture of the cylinders is shown in Figure 1.

In comparison to the previous section, $\mu_{a}$ and $\mu_{s}^{\prime }$ from measurements on cylinders instead of slabs were determined. The cylinders were made from the same material. For this purpose, the geometry of the theoretical model was changed from a slab to a cylinder lateral infinitely extended slab (see Section 2.3). To show the validity of the cylinder model, $\mu_{a}$ and $\mu_{s}^{\prime }$ of cylindrical phantoms with thicknesses of 8, 10, 12 and 14 mm are determined and are compared to the $\mu_{a}$ and $\mu_{s}^{\prime }$ of the slabs; see Figure 4. The set comprises a total of 12 phantoms across the three optical properties (Phantom P1, P2 and P3). An example photo of the cylinder phantoms with four diameters are shown in Figure 1b. Reflectance and transmittance measurements were conducted three times for each phantom, while the sample was newly positioned at the port of the integrating sphere for each measurement. The phantoms were attached to the sample port vertically and adjusted to be in the middle of the port per eye.

Figure 4 presents the results of $\mu_{a}$ and $\mu_{s}^{\prime }$ in comparison to the slab optical properties for the wavelength range of 500–1000 nm. This spectral range was selected because outside this range, the signal noise ratio of the transmittance of the 12 and 14 mm cylinders decreased strongly. Nevertheless, this wavelength range provides sufficient demonstration of the model’s applicability. The results show an excellent agreement with the optical properties $\mu_{a}$ and $\mu_{s}^{\prime }$ determined from slab measurements. Panel (a) presents the values for Phantom P1, panel (b) for Phantom P2 and panel (c) for Phantom P3. Notably, the optical properties of all thicknesses agree well. In $\mu_{a}$, there is a maximum error of around 10%, and in $\mu_{s}^{\prime }$, there is a maximum error of around 4%. The maximum mean deviation observed in comparison to slab geometry across all wavelengths in $\mu_{a}$ is 6.5% for Phantom P2 with d=10 mm, while in $\mu_{s}^{\prime }$, in it is 3.5% for Phantom P1 with d=8 mm. Small systematics in $\mu_{a}$ are observed with higher deviations at lower wavelengths which increase with increasing scatterer concentration. In general, $\mu_{s}^{\prime }$ is more accurately determined relative to the $\mu_{s}^{\prime }$ obtained from the slab measurements than is the case for $\mu_{a}$.

Although the absorption coefficient was not specifically increased in these experiments, the results convincingly demonstrate the applicability of the cylindrical model as the values of $\mu_{s}^{\prime }$ range from 0.5 to 6 mm^−1^ across four diameters. Also, both $\mu_{a}$ and $\mu_{s}^{\prime }$ show a good match to the slab $\mu_{a}$ and $\mu_{s}^{\prime }$. For phantom P3 and the cylinder with a diameter of 14 mm, the transmittance at 500 nm is below 0.2%, limiting the usable wavelength range. However, even at such low transmittance, $\mu_{a}$ and $\mu_{s}^{\prime }$ are comparable to those of other thicknesses. Consequently, the optical properties $\mu_{a}$ and $\mu_{s}^{\prime }$ of cylinders can be determined quite accurately even for low transmittance values. This is limited by the thickness in combination with the optical properties $\mu_{a}$ and $\mu_{s}^{\prime }$ of the sample. The minimum thickness of the cylinder is limited by the incident light beam diameter. Currently, the thinnest measured thickness was 8 mm, as the light source had a diameter of approximately 5 mm, making it challenging to position thinner samples. Some small systematic errors remain, which could be addressed in future work.

### 3.3. Comparison of the Optical Properties Between the Finger and Slab Phantoms

Finally, this section aims to demonstrate the applicability of the cylindrical model for a human finger. For this purpose, *R* and *T* of each finger phantom (P1, P2 and P3) were measured, and $\mu_{a}$ and $\mu_{s}^{\prime }$ were determined using the cylindrical model. The finger phantom was positioned with the palmar side facing the sample port and the cylindrical axis was oriented vertically to the horizontal axis. The illumination spot was targeted at a region between the first finger joint and the fingernail of the finger phantom. No dedicated finger holder was used; instead, the phantoms were manually centred in the port by visual inspection.

**Figure 5:**
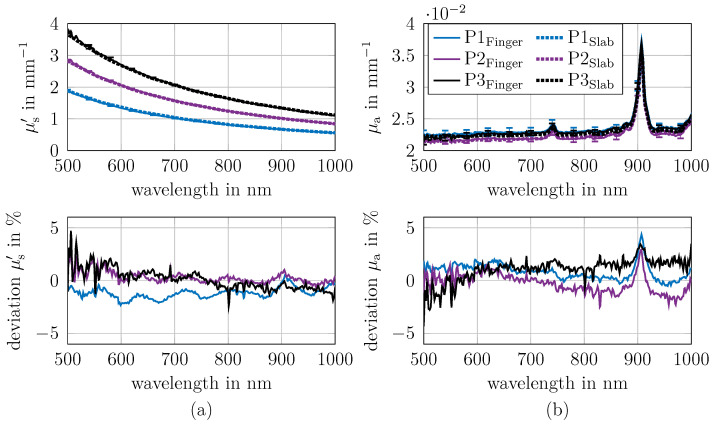
Optical properties $\mu_{s}^{\prime }$ (panel (**a**)) and $\mu_{a}$ (panel (**b**)) of the finger phantoms P1, P2 and P3 in comparison to those of the slabs. The second row shows the deviations between the slab and finger optical properties. The maximum mean error over all wavelengths is 2% in $\mu_{a}$ and $\mu_{s}^{\prime }$. An example picture of the finger phantom P1 is shown in Figure 1c.

To determine the optical properties $\mu_{a}$ and $\mu_{s}^{\prime }$ of the phantom finger, the thickness at the illumination site was measured, yielding a value of 12.45 mm. This thickness was then used to calculate $\mu_{a}$ and $\mu_{s}^{\prime }$ using the look-up table based on the cylindrical model. The extracted values for $\mu_{a}$ and $\mu_{s}^{\prime }$ for all three finger phantoms are compared in Figure 5 (top row) to those of the corresponding slab. In the bottom row, the deviations between the slab and finger optical properties are shown.

The results show a very good agreement between the $\mu_{a}$ and $\mu_{s}^{\prime }$ derived from the slab and those obtained from the finger phantoms. Specifically, the mean error across all three phantoms was below 2% for both $\mu_{s}^{\prime }$ and $\mu_{a}$. However, it is important to note that the accuracy of the optical properties of finger phantoms cannot be better than that of cylindrical phantoms, as modelling errors occur here. Therefore, it is possible that the agreement is coincidental and that the corresponding errors have not been explicitly quantified in this analysis. However, the results demonstrates that the cylindrical geometry can be applied to non-perfectly cylindrical shapes, such as a finger. In comparison to [25], where we attempted to make a phantom with the same *R* and *T* of a finger, a more accurate representation of the light source in the Monte Carlo simulation was implemented, which also takes into account the exact direction of the light source in order to reduce systematic errors. In summary, the accuracy in determining the optical properties $\mu_{a}$ and $\mu_{s}^{\prime }$ of cylinder and finger phantoms shown in this paper is within the range reported in the literature for other integrating sphere systems applied to slabs and is therefore feasible [1,11]. To determine the optical properties of even more complex geometries, a voxel-based Monte Carlo simulation could be used to model the geometries with high precision. However, positioning becomes a greater challenge for more complex shapes.

### 3.4. Optical Properties of Human Finger

In this section, $\mu_{a}$ and $\mu_{s}^{\prime }$ of five human little fingers were determined. The procedure followed was the same as described in Section 3.3. Measurements were performed three times on the region between the fingernail and the first finger joint, with the palmar side facing the integrating sphere. Between each measurement, the finger was removed and repositioned. All finger measurements were performed by the authors themselves and carried out in compliance with relevant ethical standards. The finger thickness varied from 12.25 mm to 13.8 mm, as shown in Table 1. The refractive index was assumed constant for all wavelengths to be 1.4. All five subjects were male, classified as Fitzpatrick skin type I, with an age between 27 and 60 years. The values of Finger F1 are the same as in [25].

**Figure 6:**
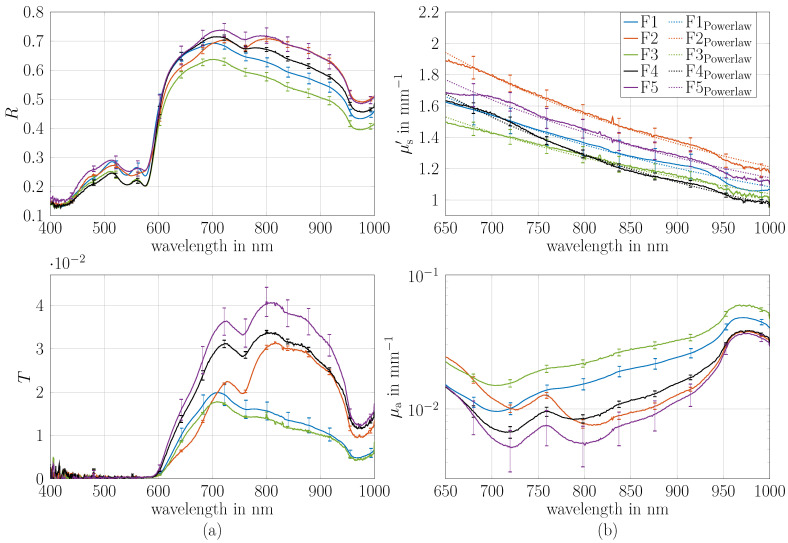
*R* and *T* values for five measured fingers from 400 nm to 1000 nm (panel (**a**)). *T*, with a good SNR, was measurable from 650 nm to 1000 nm. Therefore, the optical properties $\mu_{a}$ and $\mu_{s}^{\prime }$ of the fingers were calculated from 650 nm to 1000 nm using the homogeneous finger model (panel (**b**)).

The measured reflectance and transmittance from 400 nm to 1000 nm for all five fingers are shown in Figure 6a. Reflectance ranged approximately between 0.14 and 0.75. Transmittance up to 4% was observed between 600 and 1000 nm; effectively no transmittance was measurable for any finger between 400 nm and 600 nm. A good signal-to-noise ratio was achieved above 650 nm. Consequently, the absorption coefficient and the reduced scattering coefficient were determined in the range of 650 nm to 1000 nm, as shown in Figure 6b, assuming a homogeneous medium.

The reduced scattering coefficient for all fingers was found to lie within the same range, between 1.4 mm^−1^ and 1.8 mm^−1^ at 700 nm, which is consistent with the literature values for human tissue [23]. The resulting values for *a* and *b* are presented in Table 1. These values are consistent with the literature values shown in [23].

The absorption coefficients range from 0.006 mm^−1^ at 720 nm to 0.06 mm^−1^ at 1000 nm, with lower absorption values showing a higher standard deviation. The applicability of tissue component determination is explored in the following two sections.

### 3.5. Determination and Examination of Tissue Components

The volume concentrations of the main absorbers in each measured finger in the considered wavelength range (water, fat, collagen, deoxygenated blood and oxygenated blood) were quantitatively determined in this section. Absorption spectra for water, oxygenated and deoxygenated blood, fat and collagen were obtained from the literature; see Section 2.4 [2,19,20,21]. The fitting procedure was described in Section 2.4.

**Figure 7:**
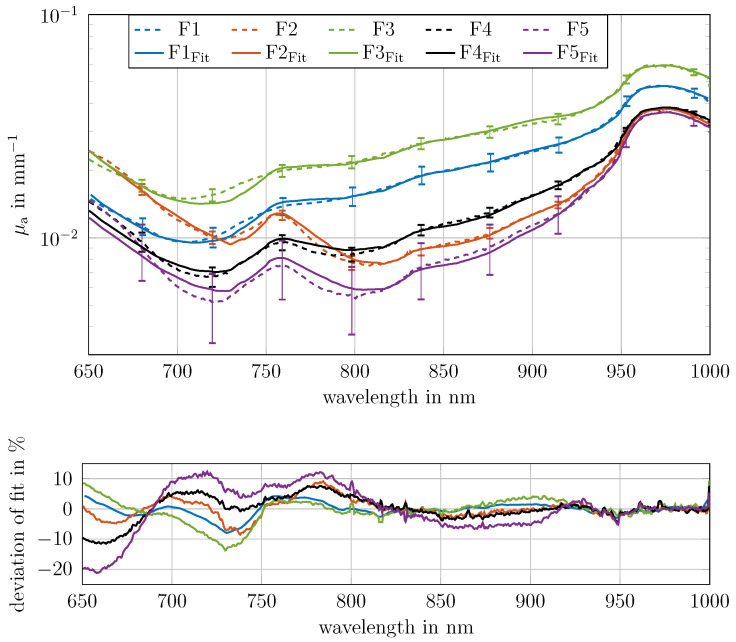
(**Top**): Absorption coefficient $\mu_{a}$ of the measured human fingers from Figure 5 and the corresponding tissue component fit using water, collagen, fat, oxy- and deoxygenated blood. The underlying individual spectra of each tissue component are shown in Figure A3. (**Bottom**): Deviations in % between the fit and the measurement.

**Figure A3:**
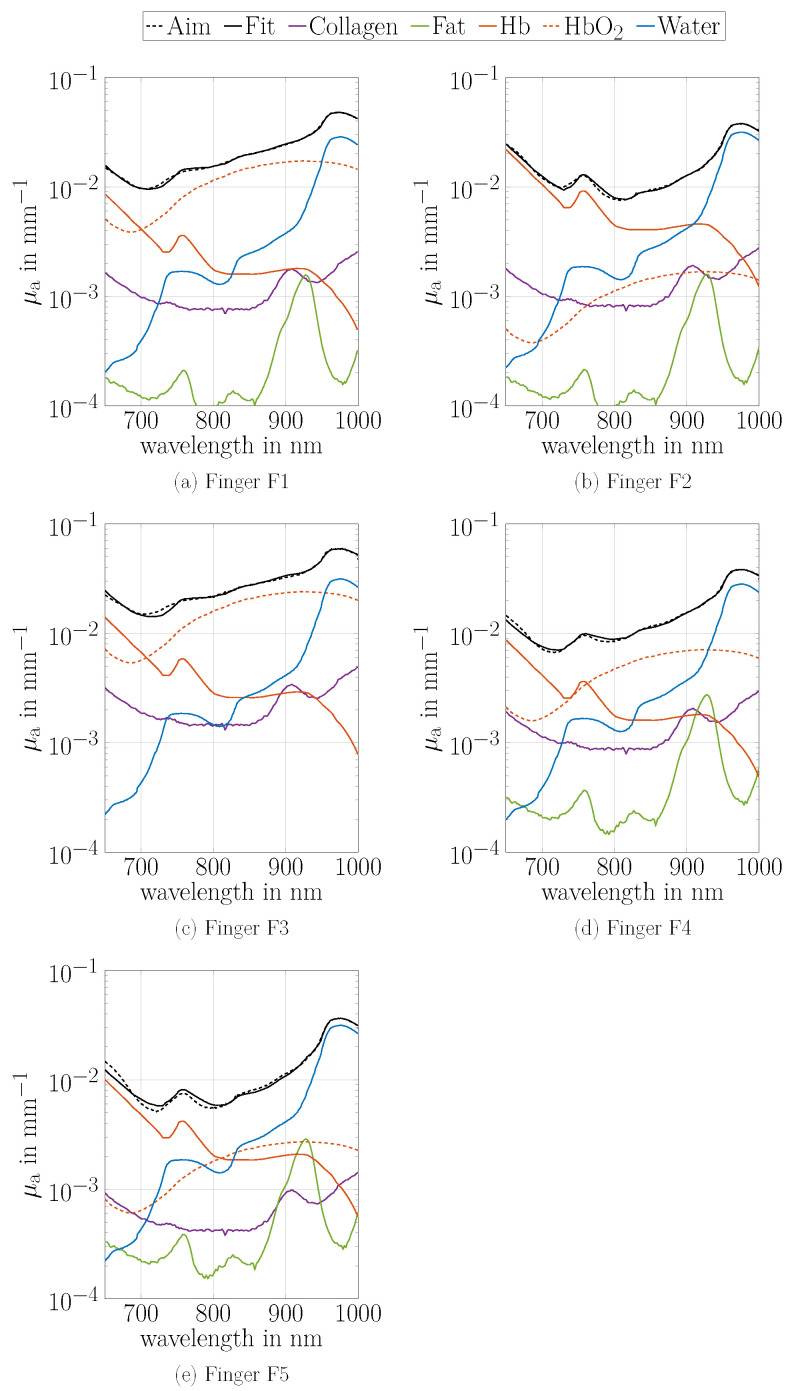
Optical properties of the individual tissue components: Water, blood (oxy- and deoxygenated), fat and collagen, weighted by the respective volume concentrations of the different fingers (1–5). The fit and the error between the fit and the measurement are shown in Figure 7.

As illustrated in Figure 7 (top row), the fitted absorption (solid line) coefficient demonstrates a strong correlation with the measured absorption coefficient (dashed line). The bottom row of Figure 7 shows the discrepancy between the fit and the measurement results. Between 650 nm and 800 nm, the relative deviations are larger for all fingers compared to the region between 800 and 1000 nm. Figure A3 shows the weighted absorption contributions of the main components. The resulting concentrations, $R^{2}$ and *E* values (see Section 2.4), are summarised in Table 2. For all data, *R*^2^ is above 0.995 and *E* is smaller 1.

Oxygen saturation ranged from 18.7% to 85.9%, total blood concentration from 0.9% to 4.3%, fat concentration from 0% to 21.0% and collagen concentration from 10.8% to 37.3%. The total volume concentrations ranged between 92.8% and 106.2%. While the boundaries of the fit allowed individual component concentrations to vary from 0 to 100%, the total concentrations for all five fingers consistently approximated 100%. This suggests that the chosen absorption spectra and tissue components represent a reasonable approach. However, these results are influenced by measurement uncertainties, similarities of the absorption spectra of the considered chromophores and variability in literature values. Variability in literature absorption values for tissue components, such as blood [19,26], collagen and fat [2], complicates exact alignment and quantification. For instance, choosing alternative literature values for blood, such as those from [26], would result in altered concentrations and different deviations. Furthermore, the spectral similarities between fat and collagen pose challenges for precise determination. The volume fractions of fat and collagen vary a lot between the fingers, but the combined fraction stays more or less the same. This suggests that these two chromophores are not very different in the spectral range that was investigated, which makes it hard to estimate them separately. Also, the absolute absorption values of fat and collagen in tissue (around 0.001 mm^−1^) are smaller than 10% of the total absorption (see Figure A3), making precise quantification difficult. The water content is nearly constant across all fingers. Fingers with lower oxygen saturation also tend to have lower blood content, which is consistent with the expected coupling between blood perfusion and oxygen supply.

Although a good fit was achieved, the higher standard deviations for low absorption values and the low absolute absorption of individual components make it difficult to reliably estimate the underlying tissue components. One potential solution to these challenges is to extend the range of wavelengths investigated and use more spectral features. This will be discussed in the next section.

### 3.6. Extended Wavelength Range Analysis

The absorption of tissue components, such as blood, exhibits distinct spectral characteristics below 650 nm (Figure 2). Extending the absorption coefficient range from 650 to 1000 nm to 400–1000 nm could enhance the determination of tissue components. This section aims to extend the wavelength range and compare the measured absorption with predictions based on tissue component concentrations (Section 3.5) to validate the determined concentrations and the proposed approach.

**Figure 8:**
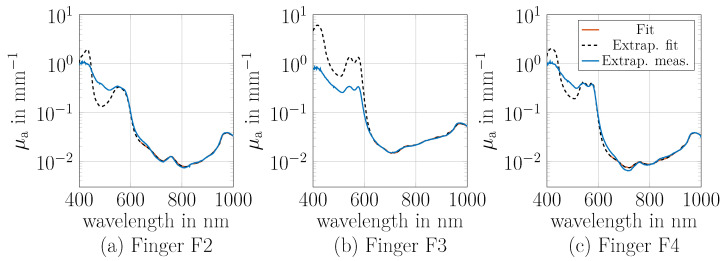
Comparison of extrapolated measured absorption coefficient from 400 nm to 1000 nm of the integrating sphere measurements with the fitted absorption coefficient obtained using the concentrations of the tissue components of Table 2 for finger F2 (**a**), F3 (**b**) and F4 (**c**). The extrapolated measured absorption coefficient was obtained by calculating $\mu_{s}^{\prime }$ from the corresponding power-law coefficients and the measured reflectance.

To extend the absorption coefficient $\mu_{a}$ to the wavelength range of 400–1000 nm, the reduced scattering coefficient $\mu_{s}^{\prime }$ was calculated for all wavelengths using the power law Equation (2). The values of the parameters *a* and *b* were taken from Table 1, assuming that these values represent the reduced scattering coefficient of the tissue across the entire wavelength range from 400 nm to 1000 nm. Based on the predefined $\mu_{s}^{\prime }$ and the measured reflectance *R* (see Figure 6), the absorption coefficient $\mu_{a}$ was calculated for the range of 400 nm to 1000 nm. The results for fingers F2, F3 and F4 are shown in blue in Figure 8 named as extrapolated measured (Extrap. Meas.). Fingers F1 and F5 demonstrate no additional effects and are thus excluded for the purpose of clarity.

The absorption coefficient resulting from the concentration-weighted absorption of the tissue components are shown in black and red. The solid red line represents the fit in the range of 650–1000 nm, as described in Section 2.4. The dashed black line indicates the predicted absorption coefficient based on the determined tissue concentrations for 400 nm to 1000 nm. Between 650 nm and 1000 nm, the calculated and fitted values exhibit good agreement, as previously shown in Figure 7. For F2 and F4, the extrapolated measured absorptions (blue line) are consistent with the predicted absorption from the tissue components (black line) down to 540 nm, while deviations occur between 400 nm and 540 nm. This suggests contributions from components such as bilirubin, carotenoids, melanin, or other unknown substances, while the determined oxygenation and total haemoglobin remain plausible. For F3, the extrapolated values fitted between 400 nm and 600 nm are significantly higher compared to the extrapolated measured data, indicating that the estimated blood concentration is primarily overestimated and that the oxygenation features also appear to differ. The observed discrepancies may be attributable to a number of factors. Firstly, the shadowing effect in cylindrical structures with high absorption, such as blood vessels, can lead to an underestimation of the absorption coefficient, particularly between 400 nm and 600 nm, due to the high absorption of blood in this wavelength range [24]. As the shadowing effect is known to increase with vessel diameter, it is evident that this effect differs from finger to finger. This is due to the fact that the vessel diameters are different for each person.

Secondly, the light penetration depth varies depending on $\mu_{a}$ and $\mu_{s}^{\prime }$ and therefore, due to the layered structure of the finger, different layers with varying tissue concentrations are observed at shorter wavelengths especially as only *R* is evaluated. With the simplified assumption that the penetration depth is proportional to $1/\sqrt{3\mu_{a}\mu_{s}^{\prime }}$, the light penetrates $\sqrt{20}$ times less deeply into the tissue for an absorption of 0.2 mm^−1^ (at 550 nm) compared to an absorption of 0.01 mm^−1^ (at 700 nm) (assuming constant $\mu_{s}^{\prime }$) [24]. Consequently, if the upper layer contains less blood, less absorption is measured at approx. 550 nm. This means that the predicted and extrapolated absorption coefficient can only match if the same volumes are probed.

Thirdly, the extrapolation of $\mu_{s}^{\prime }$ may not be entirely valid for the wavelengths from 400 nm to 650 nm, as different layers with varying $\mu_{s}^{\prime }$ are probed due to variations in penetration depth. Furthermore, the proposed power law coefficients *a* and *b* do not replicate the steep increase in $\mu_{s}^{\prime }$ with decreasing wavelengths, as already mentioned in Section 2.4, and therefore, the extrapolated $\mu_{a}$ is also determined incorrectly. With these possible influences of the shadowing effect, layered structure, which is assumed to be homogeneous, and the power law assumption, the question arises why, for the other fingers F2 and F4, these values match up to 540 nm. These findings highlight the need for further research to refine the model and account for inhomogeneous tissue structures. For example, a simulation-based evaluation for the integrating sphere or the use of additional measurement methods, such as time-resolved techniques or spatial frequency domain (SFD) imaging, could provide deeper insights into these effects and help address the current limitations.

## 4. Conclusions

This study analyses the feasibility of determining the optical properties $\mu_{a}$ and $\mu_{s}^{\prime }$ of a cylinder using an integrating sphere and its potential application to a human finger. For this purpose, the optical properties $\mu_{a}$ and $\mu_{s}^{\prime }$ of slabs, cylindrical phantoms and finger-shaped phantoms were determined and compared. Finally, $\mu_{a}$ and $\mu_{s}^{\prime }$ of five human little fingers were determined and the tissue components, oxygenated and deoxygenated blood, water, and fat and collagen, were determined.

First, $\mu_{a}$ and $\mu_{s}^{\prime }$ were determined for the slabs using an already established evaluation process [6]. These values were then validated by comparing them with predictions based on the weighted mass concentrations of the components. Subsequently, $\mu_{a}$ and $\mu_{s}^{\prime }$ of the cylinder phantoms were determined and confirmed by comparison with those of the slab. To demonstrate the applicability of the cylindrical model to fingers, the determined $\mu_{a}$ and $\mu_{s}^{\prime }$ of the phantom fingers were then compared with those of the slabs. For all cylinders and fingers, there was a very good agreement in $\mu_{a}$ and $\mu_{s}^{\prime }$ with those of the slabs.

The optical properties $\mu_{a}$ and $\mu_{s}^{\prime }$ of five fingers determined here could serve as the basis for developing further finger phantoms. A phantom-based investigation of the photoplethysmography signal has been published recently in the literature [27,28,29,30]. So far, literature values for the optical properties of the dermis have been used for this purpose. As the finger only consists of a small part of dermis and the investigated individuals are classified as Fitzpatrick skin type I, the determined $\mu_{a}$ and $\mu_{s}^{\prime }$ could be utilised for a homogeneous finger. Furthermore, the optical properties could be applied to prosthetics in the visible range or for testing fingerprint sensors.

It was demonstrated that tissue components, de-/oxygenated blood, fat, collagen and water, can be determined using the absorption in the wavelength range from 650 nm to 1000 nm as the fitted spectrum aligned well and the determined tissue component concentrations appear plausible, as presented in Table 2. A trend was identified between oxygen saturation and total blood content, whereby lower oxygen saturation was concomitant with reduced blood concentration, in accordance with physiological expectations. Fat and collagen contribute less than 10% to the determined $\mu_{a}$, which limits their diagnostic significance. Further work could address a comparison with pulse oximetry, particularly to evaluate differences between oxygen saturation measured by pulse oximetry and tissue oxygen saturation of the whole blood in the finger (also non-pulsatile), potentially enabling deeper clinical insights. In addition to the analysis of the absorption coefficient, subsequent research could further explore the spectral dependence of the reduced scattering coefficient $\mu_{s}^{\prime }$. Variations in the short-wavelength region may offer insights into tissue microstructure and collagen content. Furthermore, the reduced scattering coefficient may offer potential for the development of optical biomarkers for the assessment of bone-related diseases, including osteoporosis.

Extending the wavelength range to 400–650 nm, as shown in Section 3.6, provides additional spectral features and valuable information. An extension beyond 1000 nm could lead to a more precise determination of the fat content. However, there are some open questions such as the influence of inhomogeneous optical properties, e.g., due to a layered tissue structure, shadowing effects or validity of the determined power law coefficient for small wavelengths. The influence of multilayer systems on integrating sphere measurements could be further investigated theoretically or using complementary measurement techniques such as spatial frequency domain imaging, time-resolved or spatially resolved measurements. Combining various measurement systems could provide a deeper understanding of the finger’s complex multilayer structure and help clarify the effects observed in Figure 8. With this knowledge, a more comprehensive understanding of clinical applications could be achieved.

This study demonstrates that integrating sphere measurements can accurately determine the optical properties of cylindrical and finger-like structures. Despite measurement uncertainties and modelling limitations, concentration of the tissue components were determined. This enhanced understanding of tissue serves as a basis for further investigations and represents a step toward improved measurement techniques.

## Figures and Tables

**Table 1:** Finger thickness, assumed refractive index and coefficients *a* and *b* for the power law of the reduced scattering coefficient (Equation (2)). Each finger was measured three times.

Sample Name	Thickness	Refractive Index	Repetition	Power Law Fit (Equation (2))
Finger F1	13.8 mm	1.4	3	*a* = 2.15 mm^−1^, *b* = 0.99
Finger F2	12.45 mm	1.4	3	*a* = 2.59 mm^−1^, *b* = 1.10
Finger F3	13.2 mm	1.4	3	*a* = 1.93 mm^−1^, *b* = 0.89
Finger F4	12.9 mm	1.4	3	*a* = 2.34 mm^−1^, *b* = 1.26
Finger F5	12.25 mm	1.4	3	*a* = 2.31 mm^−1^, *b* = 1.01

**Table 2:** Determined tissue components of the measured fingers, including water, fat, collagen, deoxygenated blood (Hb) and oxygenated blood (HbO_2_) in volume percent. Oxygenation saturation (SO_2_) was calculated from HbO_2_/(Hb + HbO_2_). Additionally, the parameters $R^{2}$ and *E* (Equation (1)) are presented.

Sample Name	Water in %	Fat in %	Collagen in %	Hb in %	HbO_2_ in %	SO_2_ in %	*R* ^2^	*E*
Finger F1	58.8	11.5	19.5	0.43	2.61	85.9	0.999	0.12
Finger F2	65.0	11.7	20.9	1.09	0.25	18.7	0.998	0.74
Finger F3	64.6	0	37.3	0.69	3.62	84.0	0.996	0.67
Finger F4	58.0	20.1	22.5	0.43	1.07	71.3	0.998	0.60
Finger F5	64.7	21.0	10.8	0.50	0.41	45.1	0.995	0.12

## Data Availability

The data supporting the conclusions of this article will be made available by the authors up on reasonable request.
